# Predicting others’ intention involves motor resonance: EMG evidence from 6- and 9-month-old infants

**DOI:** 10.1016/j.dcn.2013.10.004

**Published:** 2013-11-01

**Authors:** Elena Natale, Irene Senna, Nadia Bolognini, Ermanno Quadrelli, Margaret Addabbo, Viola Macchi Cassia, Chiara Turati

**Affiliations:** Università degli Studi di Milano-Bicocca, Italy

**Keywords:** Motor resonance, Action understanding, Grasping skills, Infancy, Cognitive development

## Abstract

The study explores infants’ ability to generate on-line predictions about others’ action goals through the recruitment of motor resonance mechanisms. To this aim, electromyography was recorded from mouth-opening suprahyoid muscles (SM) of 9-month-old infants while watching a video of an adult agent reaching-to-grasp an object and bringing it either to mouth or head. The results demonstrated, for the first time, that at the age of 9 months there is a dynamic mirror modulation of SM activity by action observation, with the infant's muscles responsible for the action final goal being recruited from the action outset. The comparison with the responses of 6-month-olds tested on the same task showed that in younger and older infants there is a different chronometry of the SM activation with respect to the different phases of the observed action (i.e., bringing vs. grasping, respectively).

Results suggest that motor resonance mechanisms triggered within the infants’ motor system by action observation undergo gradual development during the first year of life. They also indicate that motor resonance may reflect anticipation of the agent's intention based on the goal of the action.

## Highlights

• Infants’ motor system is recruited by action observation.
• Infants’ motor system is involved in the anticipation of the action goal.
• Infants’ motor experience affects the ability to predict the action goal.
• Motor resonance mechanisms gradually develop during the first year of life.

## 1 Introduction

The ability to understand and attend prospectively to others’ actions is crucial to social life. Behavioral evidence has indicated that in humans such ability develops during the first years of life. By three years of age, children can generate and verbally report predictions about actions based on intentions, desires and knowledge states (Wellman, 1992, Wimmer and Perner, 1983). This capacity has been recently observed even in non-verbal toddlers through the second year of life by using non-verbal measures such as looking times (Onishi and Baillargeon, 2005, Southgate et al., 2007, Surian et al., 2007). Using paradigms that capitalize on infants’ looking behavior, evidence has also been provided showing that the ability to perceive others’ actions in terms of their goal is present by the end of the first year of life. For instance, after habituation to a goal-directed action infants subsequently look longer at test events in which the action goal is altered than at events in which the goal is preserved and the physical properties of the action are changed (Biro and Leslie, 2007, Woodward, 1998, Woodward, 2009). In a similar vein, infants anticipate the goal of others’ action by performing proactive eye movements toward the spatial position within the scene that corresponds to the action's end state (Falck-Ytter et al., 2006, Gredebäck et al., 2009, Kanakogi and Itakura, 2011, Kochukhova and Gredebäck, 2010). For instance, when observing a hand placing objects into a container, 1-year-old infants make eye movements toward the container before the hand reaches it (Falck-Ytter et al., 2006). Eye-movement anticipation has been observed even in 11-month-old infants by using a modified version of the classical Woodward's (1998) paradigm, in which goal and hand movement patterns were not confounded (Cannon and Woodward, 2012).

Notwithstanding the relevance of the above-mentioned literature on action perception and comprehension, the issue of whether and how infants are able to generate on-line predictions about others’ action goal remains open. Indeed, recently the question has been raised of whether adults’ proactive eye movements would truly reflect the capacity to anticipate others’ action goal (Eshuis et al., 2009). Moreover, despite infants’ ability to attribute goals to others’ action has been extensively examined by means of behavioral paradigms, its neurophysiological underpinnings have been poorly investigated and still need to be fully understood.

By using electroencephalography (i.e., EEG, Southgate et al., 2010, Stapel et al., 2010) and electromyography (i.e., EMG, Turati et al., 2013), it was shown that the infants’ motor system is recruited during the observation of goal-directed actions, suggesting the existence of mirror-like motor neural mechanisms in early infancy. In particular, Turati et al. (2013) recorded surface EMG activity from mouth-opening suprahyoid muscles (i.e., SM) of 3- and 6-month-olds, while infants were watching videos of an actress performing an object-to-mouth and an object-to-head action, with both actions including the same movement phases (i.e., reaching for, grasping, bringing). Actress's SM are specifically engaged in the achievement of the final goal of the object-to-mouth, but not the object-to-head action. By recording infants’ SM activation during action observation, the authors provided the first direct demonstration that infants’ muscle activation is on-line modulated by action observation, hence documenting the presence of a motor resonance effect. Specifically, it was shown an increase of 6-month-olds’ SM activation during observation of the object-to-mouth action as compared to the object-to-head action, whereas no motor resonance effect was observed in 3-month-olds. Importantly, in 6-month-old infants the motor resonance effect was found to selectively arise during observation of the latest phase of the action (i.e., the bringing phase) where the action goal is achieved, whereas no modulation of SM activity was found during earlier action phases (i.e., the reaching for and grasping the object phases). These findings indicate that from 6 months of age infants manifest an automatic simulation of the observed action, which may support their appreciation and understanding of the action goal. However, unlike 5–9 year-old children, showing a modulation of mylohyoid muscle (i.e., one muscle of the SM group) activity already during earlier phases of the grasping action (Cattaneo et al., 2007), 6-month-olds were not able to on-line predict the agent's goal. Turati et al. (2013) proposed that such delay in EMG modulation in 6-month-olds as compared to children might reflect a developmental progression from a motor resonance mechanism triggered by the observation of the action goal to one driving goal anticipation.

The current study aimed at further exploring the development of mirror motor mechanisms by testing 9-month-old infants with the same EMG paradigm as that employed with younger infants (Turati et al., 2013). We sought to find out whether, unlike in younger infants (Turati et al., 2013) but similarly as in children (Cattaneo et al., 2007), in 9-month-olds the muscles responsible for the action final goal increase their activity as soon as the action starts. To this aim, following Cattaneo et al. (2007), we analyzed the 9-month-old infants’ SM activation elicited by action observation as a function of the three movement phases of reaching for, grasping, and bringing the object to mouth or head. In an additional analysis, 9-month-olds’ performance was also compared with that of 6-month-olds (Turati et al., 2013).

## 2 Methods

### 2.1 Participants

Thirty healthy, full-term 9-month-olds (19 females, mean age = 9 months 2 days, range = 259–290 days) were randomly assigned to two different experimental groups: 15 (8 females, mean age = 9 months and 2 days, range = 262–288 days) to Group 1 and 15 (11 females, mean age = 9 months and 1 days, range = 259–290 days) to Group 2. Thirteen infants were also tested, but then discharged from the experimental groups because of fussiness and no completion of the minimum number of trials required for data analysis. Data from the 9-month-olds tested in the present study were compared to those obtained by Turati et al. (2013) with 6-month-old infants. The final sample in Turati et al.’s study was composed of 30 infants, 17 (7 females, mean age = 6 months and 9 days) assigned to Group 1 and 13 (5 females, mean age = 6 months and 8 days) to Group 2. Infants in each group were tested with the same experimental procedure as in the current study.

The protocol was carried out in accordance with the ethical standards of the Declaration of Helsinki (BMJ 1991; 302: 1194) and approved by the ethical committee of the University of Milano-Bicocca. Parents gave their written informed consent.

### 2.2 Stimuli, apparatus and procedure

**Fig. 1:**
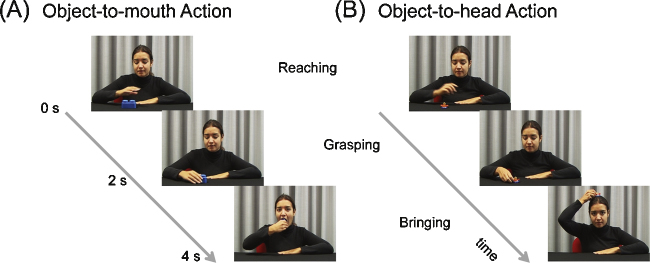
A schematic illustration of the videos shown to infants, with the crucial frames of the (A) object-to-mouth action and (B) object-to-head action.

EMG activity was recorded from SM during the observation of videos depicting an actress reaching for, grasping and bringing either an object to the mouth (object-to-mouth action) or an object to the head (object-to-head action) (Fig. 1). Each infant was shown the two actions, which were directed to different objects, i.e. a pacifier and a piece of lego, both visible throughout the whole video. Thereby, the object represented a cue to understand which was the goal of the observed action. In order to control for possible effects of the displayed object, participants were assigned to one of two different groups: infants in Group 1 were shown the actress bringing a pacifier to the mouth and a piece of lego to the head; infants in Group 2 watched the actress bringing the piece of lego to the mouth and the pacifier to the head.

The experiment took place in an audiometric cabin equipped with a Faraday cage, where participants seated in an infant seat ∼60 cm from a 24-in. screen. Each trial began with an animated fixation point. As soon as the infant looked at it, the experimenter started the video, lasting 4 s and consisting of 100 frames, 40 ms each. In particular, for both actions, frame 51 (i.e. 2 s from the video onset) depicted the exact moment in which the actress's hand touched the object. Distinct phases of the actress's movement, i.e., reaching for, grasping and bringing the object had the same duration across the two different actions. We defined the three phases as following: the reaching phase lasted from the beginning of the video until the moment in which the hand started to shape into a grasp (duration = 1.6 s); the first frame in which the hand started to shape into a grasp signaled the beginning of the grasping phase (duration = 0.8 s), and the first frame in which the object was lifted signaled the beginning of the bringing phase (1.6 s). In the object-to-mouth action, the actress opened her mouth during the phase of bringing, slightly before the object got in contact with her mouth. At the end of the video, a colored circle slowly expanding and contracting was displayed for 3.5 s, followed by a 500 ms blank screen. The two types of actions were presented in alternated blocks of trials and in a sequence counterbalanced between subjects. There was no restriction in number of blocks or trials shown, i.e., they could be played indefinitely. However, when infants reached the criterion of watching at least 70% of the video across five trials, the block ended and the subsequent block of trials was shown. The experimental session was terminated when infants looked away for at least 2 s during each of five consecutive trials. On average, 16.5 trials (range = 5–37) were presented for each action type. The number of presented trials was not significantly different for the two types of action (*p* > .7). The computer controlled the sequence and timing of the stimuli.

A questionnaire was administered to the parents to assess infants’ familiarity with the objects displayed in the video and their grasping skills. The questionnaire was originally devised by Turati et al. (2013), who administered it to the parents of the 6-month-old infants participating in their study, without however reporting the data in their original manuscript. On the questionnaire items about familiarity with the objects, parents were asked to indicate whether or not their infant used the pacifier and whether or not he/she had any experience as a player with building blocks toys. Infants using and not using the pacifier were scored as 1 and 0, respectively. Infants with and without experience with building blocks were scored as 1 and 0, respectively. The item about grasping abilities included pictures depicting the different types of grasp: the raking grasp, involving all fingers but thumb to wrap around and hold an object; the palmar grasp, using all fingers against the palm to do the holding; the pincer grasp, using index, medium fingers and thumb to pick up an object. Parents were asked to indicate what type of grasp their infant was currently able to perform among those shown in the pictures. When parents indicated more than one category, the higher score was attributed, corresponding to the more developed type of grasp. Raking, palmar and pincer grasps were scored as 0, 1 and 2, respectively.

### 2.3 EMG recording, signal processing and data analysis

A Digitimer electromyograph was used to record the EMG signal from the infants’ SM. Two surface electrodes for pediatric use were placed 2 cm apart under the infant's chin symmetrically to the midline. The reference electrode was positioned ∼2 cm above the nasion. The EMG signal was amplified (gain 1000), filtered (band-pass: 10–1.000 Hz), sampled at 1 kHz, and stored for offline filtering (150 Hz; high-pass: 30 Hz). Impedance was between 5 and 10 kΩ. The EMG signal was then rectified for analysis.

Infants’ looking time toward the stimuli was coded on-line and trials in which infants looked at the video for less than 70% of its duration were discarded. Looking time was also coded off-line by a second observer. Pearson correlation revealed a high degree of agreement between the two coders on the trials to be discarded based on the looking time criterion, *r* (60) = .99, *p* < .0001. In order to avoid any spurious effect produced by infants’ movements while watching the videos, trials were also discarded off-line whenever signal noise and motion artifacts contaminated the recordings. As a consequence, about 48% of object-to-mouth and object-to-head action trials were excluded from data processing. Only infants with at least 4 trials per action type were included in the analyses. On average, each infant contributed to the analyses with 8 trials (range = 4–18) per action type. The number of trials included in the analysis did not significantly differ for the two types of action (all *ps* > .4).

Following Cattaneo et al. (2007), the EMG signal recorded during the 4-s video presentation was segmented into three epochs, each corresponding to a distinct phase of the action: epoch 1, corresponding to the reaching phase (duration = 1.6 s), epoch 2, corresponding to the grasping phase (duration = .8 s), and epoch 3, corresponding to the bringing phase (duration = 1.6 s). The area under the curve of the rectified EMG activity was computed separately for every epoch on a trial-by-trial basis, normalized (*z*-scores), and then averaged for each type of action.

## 3 Results

### 3.1 Muscle activation during action observation in 9-month-old infants

A repeated-measures Analysis of Variance (ANOVA) on the EMG signal (*z*-scores) with action (object-to-mouth vs. object-to-head action) and epoch (epoch 1 vs. epoch 2 vs. epoch 3) as within-subjects factors revealed a significant action by epoch interaction, *F*(2, 58) = 3.52, *p* = .03, whereas no significant main effects were found (all *ps* > .2). Post hoc t-tests showed that for the object-to-mouth action 9-month-olds’ SM activation was greater during the grasping than the bringing phase, *t*(29) = −2.7, *p* = .01, and it also tended to be greater during the grasping than the reaching phase, *t*(29) = 1.9, *p* = .06. As to the object-to-head action, SM activity recorded from 9-month-old infants did not change across the three action phases (all *ps* > .2). Finally, 9-month-olds had a greater SM activation during the grasping phase of the object-to-mouth than that of the object-to-head action, *t*(29) = 2.1, *p* = .04. No other comparisons were found to be significant, all *ps* > .2.

To verify whether 9-month-olds’ SM response might change with the object of the bringing-to-mouth action, a repeated-measures ANOVA on the EMG signal recorded during the bringing-to-mouth action with epoch (epoch 1 vs. epoch 2 vs. epoch 3) as within-subjects factor and object (pacifier for the Group 1 vs. piece of lego for the Group 2) as between-subjects factor was carried out. The analysis revealed only a main effect of the epoch, *F*(2, 56) = 3.9, *p* = .02, whereas no significant main effect of the object as well as significant epoch by object interaction were found, all *ps* > .5.

### 3.2 Motor resonance effects in 6- and 9-month-old infants

To better substantiate how motor resonance effects triggered by action observation develop during the first year of life, we directly compared SM activity observed in the current sample of 9-month-olds with SM activity recorded from the thirty 6-month-old infants tested by Turati et al. (2013) (see Section 2.1). Because in Turati et al.’s study the EMG signal was segmented in two epochs (i.e., reaching for and grasping the object vs. bringing the object to mouth or head), we re-analyzed the original 6-month-olds’ data collected by Turati and colleagues by segmenting the signal into three epochs, as described above. The 6-month-old infants (i.e., Group 1, *N* = 17, 7 females, mean age = 6 months and 9 days; Group 2, *N* = 13, 5 females, mean age = 6 months and 8 days) were previously tested using the same experimental procedure as done here (see Turati et al., 2013 for details).

The EMG data (*z*-scores) were then analyzed via a repeated-measures ANOVA with age (6 vs. 9 months) as between-subjects factor, and action and epoch as within-subjects factors. Only a significant age by action by epoch interaction emerged, *F*(2,116) = 7.49, *p* < .001; no other effects reached significance, all *ps* > .1. The 3-way interaction was explored with two separate ANOVAs, one for each type of action, with age as between-subjects factor and epoch as within-subjects factor. For the object-to-mouth action, there was a significant age by epoch interaction, *F*(2,116) = 6.11, *p* < .01, with no significant main effects, all *ps* > .3. Post hoc paired samples t-tests indicated that 6-month-olds showed greater SM activation during the bringing phase compared to the grasping phase, *t*(29) = −2.3, *p* = .02. No other comparisons reached significance, all *ps* > .2. In contrast, as reported above 9-month-olds showed greater SM activation during the grasping as compared to the other phases of the action.

**Fig. 2:**
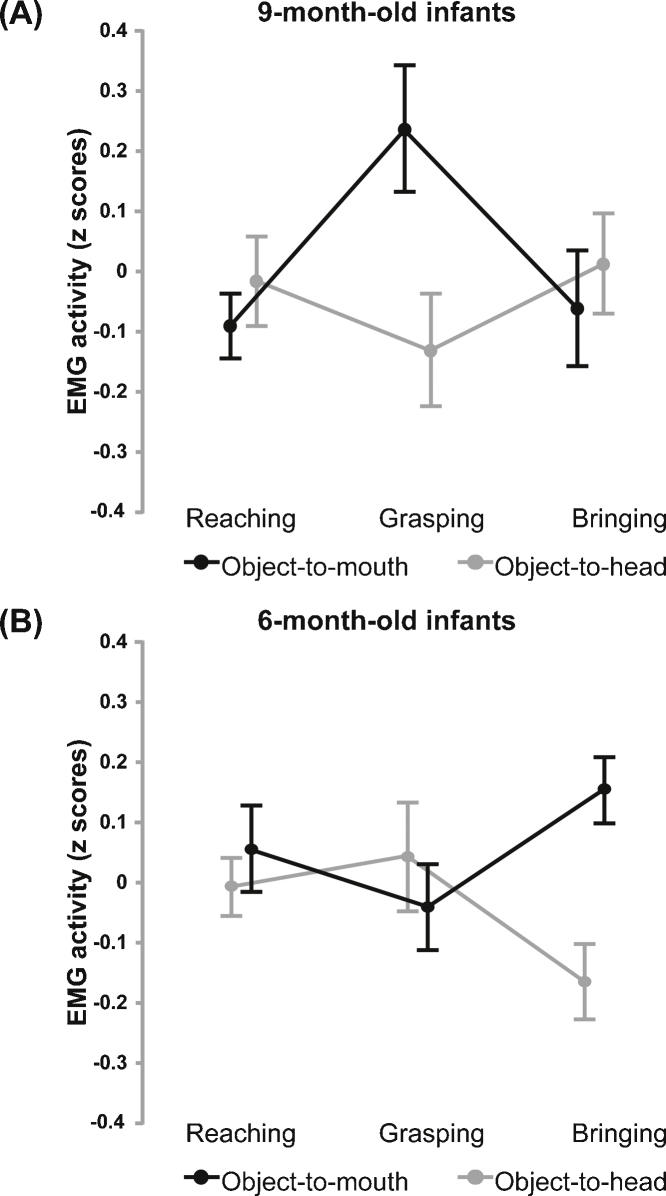
SM activation of (A) 9-month-old and (B) 6-month-old infants during action observation plotted as a function of the movement phase, i.e. reaching for, grasping, and bringing, and the type of action, i.e. object-to-mouth (black lines) and object-to-head (gray lines) action. Error bars = SEM.

For the object-to-head action, the age by epoch interaction approached significance, *F*(2,116) = 2.78, *p* = .06, whereas no main effects were found, all *ps* > .6. Post hoc paired samples *t*-tests showed that, unlike 9-month-olds’ SM activity which was not modulated by the action phase, 6-month-olds’ SM activation during the bringing phase was significantly smaller than during the reaching phase, *t*(29) = 2.5, *p* = .01, and marginally smaller than during the grasping phase (*p* = .08), with no other comparisons reaching statistical significance (*p* > .6) (see Fig. 2).

### 3.3 Familiarity with the object of observed actions and grasping skills

On the item of the questionnaire related to the infant's experience with the pacifier, thirteen 6-month-olds and ten 9-month-olds were scored 0, whereas seventeen 6-month-olds and twenty 9-month-olds were scored 1. On the item asking whether infants have played with building blocks toys, twenty-five 6-month-olds and seventeen 9-month-olds were scored 0, whereas five 6-month-olds and thirteen 6-month-olds were scored 1. A Mann–Whitney *U*-test was separately carried out on the score obtained in these items with age (6- vs. 9-month-olds) as between-subjects factor. The analyses revealed that 6- and 9-month-olds had a similar score for their experience with pacifier, *U* = 405, *z* = −0.78, *p* = .4, whereas 6-month-olds were scored lower than 9-month-olds for the experience with building blocks toys, *U* = 330, *z* = −2.2, *p* = .02. Moreover, the EMG data were re-analysed by separately considering the score at each item of the questionnaire and running two ANOVAs with age (6 vs. 9 months) and experience (yes vs. no) as between-subjects factors, and action (object-to-mouth vs. object-to-head action) and epoch (epoch 1 vs. epoch 2 vs. epoch 3) as within-subjects factors. Both the analyses revealed only a significant age by action by epoch interaction, *ps* < .001. No other effects reached significance, all *ps* > .08.

At the assessment of grasping skills most of 9-month-olds (*N* = 23) obtained a score of 2, indicating that they had developed the ability to perform pincer grasps; five 9-month-olds obtained a score of 1 (i.e., palmar grasp), and only two infants obtained a score of 0 (i.e., raking grasp). Six-month-old infants (*N* = 25) in Turati et al. (2013) were mainly scored 1; three obtained a score of 2 and two were scored 0. A Mann–Whitney *U*-test on the grasping score with the between-subjects factor age (6- vs. 9-month-olds) confirmed that 9-month-old infants were scored higher for their motor grasping abilities in comparison to 6-month-olds, *U* = 170, *z* = −4.6, *p* < .001. However, being age and grasping skills variables almost collinear the possible effect of the latter on motor resonance triggered by action observation was not further explored.

## 4 Discussion

In the present study, surface EMG was recorded from 9-month-olds during action observation in order to verify whether at this age motor resonance may reflect the ability to anticipate the action's goal, similarly as observed in children.

To this aim, following Turati et al. (2013) the activity of muscles typically active during mastication and swallowing (i.e. suprahyoid muscles, SM) was recorded while infants looked at videos displaying an adult agent reaching for, grasping and bringing an object either to mouth or to head. The novel finding is that, unlike 6-month-old infants (Turati et al., 2013) 9-month-olds show early motor resonance effect, arising during observation of the actress grasping the object to bring it to mouth. This result provides strong evidence that at the age of 9 months mirror motor mechanisms are fully active during action observation, with the infant's muscles responsible for the action final goal being recruited from the action outset. Importantly, Cattaneo and colleagues (2007, see their Experiment 1) have reported an overall increase of Typically Development (TD) children's EMG activity during observation of the eating as compared to the placing action, whereas in 9-month-olds we found a significant modulation of activation with the action selectively arising during the grasping phase. Beyond the different age of participants, such a discrepancy is likely to depend on the experimental setting and the criteria to define epochs. In particular, in Cattaneo et al. (2007) children observed a real actor always bringing some food to mouth, whereas in our study infants watched videos of an actor bringing one of two non-edible objects to mouth. Therefore, in the bringing-to-mouth action stimuli might have been somehow more engaging for children than infants, yielding an overall greater effect in the former than the latter group. Also, here the grasping phase started when the hand began to shape into a grasp rather than later on (i.e. from the contact with the touch-sensitive plate on which the food was placed) as in Cattaneo et al. (2007). While enabling us to evaluate EMG activation during a relatively large time window, which is more suitable for studying infants, the longer duration of our grasping phase (0.8 s) as compared to that (0.22 s) of Cattaneo et al. (2007) might have contributed to the discrepancy in results between the two studies. Specifically, in our study the EMG activation run out during the grasping phase, instead in Cattaneo et al. (2007) it persisted during the bringing phase. Nevertheless, it is important to note that the visual inspection of the time course of EMG activity as reported by Cattaneo et al. (2007) for TD children reveals that the observers’ EMG activation tended to increase progressively from 2 s prior up to the moment in which the agent grasps the food (time 0) and, conversely, appeared to decrease from time 0 to 2 s afterwards, suggesting a similar trend as that found in our study for 9-month-old infants.

The comparison between 9- and 6-month-olds tested in a previous study (Turati et al., 2013) indicates a clear-cut difference in the action phase triggering the motor resonance effect at different ages. Specifically, although at both ages there is a dynamic modulation of SM activity during the observation of different action's phases, 9-month-old infants showed an enhancement of SM activity that was specific for the grasping phase of the object-to-mouth action. Instead, in 6-month-old infants SM activation was enhanced on a later time window, during the bring phase of the object-to-mouth action. On the other hand, a different pattern of SM activation was found during the observation of the object-to-head action: under this condition, in 9-month-old infants the SM activity was not modulated by the action phase, while in 6-month-olds there was a reduction of SM activation during the bring phase as compared to the reach phase. All together, present evidence suggests that mirror mechanisms gradually develop: at 6 months of age, the motor system is recruited only when the action goal is achieved, rather than before achieving the goal as in 9-month-old infants. Hence, our findings indicate that active, motor mirror mechanism may not contribute to a full comprehension of the intention of others before 9 months of age. Noteworthy, previous evidence also showed that such mirror resonance effects completely lack in 3-month-old infants (Turati et al., 2013).

The early engagement of mirror mechanisms during action observation supports the idea that they may allow 9-month-old infants to immediately capture the intention of the agent. Importantly, our physiological data shed light into the possible functional and neural mechanisms underlying action prediction. Indeed, muscle activation prior to the observation of the movement phase in which the agent's muscle would be active can be taken as a direct evidence that, similarly as children, infants anticipate others’ intention based on the goal structure of the action (see also Cannon and Woodward, 2012 for a critical discussion about this issue). This is in line with the hypothesis that the motor system would play an active role in generating predictions about the end state of the observed actions, rather than passively reflecting the observed action (see Kilner et al., 2004, Kilner et al., 2007, Southgate et al., 2009).

In the present study, we have also verified whether the observed motor resonance effects might depend on infants’ familiarity with the objects of the agent's action (pacifier and lego), as assessed by means of a questionnaire administered to the parents. Familiarity with objects did not affect EMG response driven by action observation, suggesting that the degree of sensorimotor experience specifically related to the objects of the action did not influence the observed motor resonance effects. Nevertheless, it is important to note that the specific nature of the action modulates infants’ ability to show proactive goal-directed eye movements. For example, Kochukhova and Gredebäck's (2010) showed that 6-month-olds may anticipate the goal of a familiar action like feeding, but not the goal of unfamiliar action like combing. Similarly, Falck-Ytter et al. (2006) demonstrated that 6-month-old infants, unlike 12-month-olds, did not anticipate the goal of placing actions.

In line with classic observation (i.e. Halverson, 1931), we found a clear-cut difference in grasping skills between 6- and 9-month-olds, with the former mostly having less developed skills (i.e. raking and palmar grasp) and the latter mostly having fine motor skills (i.e. pincer grasp). Although we could not test for the effect of grasping abilities independently from age differences on EMG response, thus providing direct neurophysiological evidence for experience-dependent motor resonance early in development (see van Elk et al., 2008 for EEG evidence from older infants), our findings suggest that infants’ sensorimotor grasping experience might affect the ability to predict the goal of the observed grasping action, highlighting a functional role for the motor system in the perception of others’ intention. In particular, developing fine motor abilities might increase infant's capacity to encode and discern a meaningful structure in other's action, which, in turn, supports action prediction. This would be in line with the proposal that infants’ own experience as intentional agents would shape their sensitivity to and understanding of others’ action (e.g. Gerson and Woodward, 2013, Sommerville et al., 2005, Sommerville et al., 2008, Libertus and Needham, 2010).

Overall, obtained evidence is consistent with the proposal that developments in action perception and action production are linked by shared neurocognitive representations (Decety and Sommerville, 2003, Lepage and Théoret, 2006, Meltzoff, 2005). Moreover, the early activation of the motor system in response to others’ action observation strongly argues in favor of the presence, early in life, of mirror motor simulation mechanisms, which may be crucially involved in predicting others’ action goal. However, further studies are needed to disentangle the relative contribution of general maturational processes and sensorimotor experience in the early emergence of motor resonance effects.

## Conflict of interest statement

We certify that there are no affiliations with or involvement in any organization or entity with a direct financial interest in the subject matter or materials discussed in the manuscript (e.g. employment, consultancies, stock ownership, honoraria, and/or expert testimony).
